# Increased COVID-19 Vaccination Hesitancy and Health Awareness amid COVID-19 Vaccinations Programs in Israel

**DOI:** 10.3390/ijerph18073804

**Published:** 2021-04-06

**Authors:** Maayan Shacham, Lee Greenblatt-Kimron, Yaira Hamama-Raz, Leslie R. Martin, Oren Peleg, Menachem Ben-Ezra, Eitan Mijiritsky

**Affiliations:** 1 School of Social Work, Ariel University, Ariel 40700, Israel; Drmaayanshacham@gmail.com (M.S.); leegreenkim@gmail.com (L.G.-K.); yhr2808@gmail.com (Y.H.-R.); menbe@ariel.ac.il (M.B.-E.); 2 Department of Psychology, La Sierra University, Riverside, CA 92515, USA; lmartin@lasierra.edu; 3 Tel-Aviv Sourasky Medical Center, Department of Otolaryngology, Head and Neck and Maxillofacial Surgery, Sackler Faculty of Medicine, Tel Aviv 6139001, Israel; orenpeleg@gmail.com; 4 The Maurice and Gabriela Goldschleger School of Dental Medicine, Tel Aviv University, Tel Aviv 6997801, Israel

**Keywords:** vaccination, vaccine hesitancy, dentists, dental hygienists, vaccination attitudes, COVID-19, SARS-CoV-2

## Abstract

In January 2021, Israel started vaccinating healthcare workers (HCWs) and individuals older than 65 years with COVID-19 vaccines. Scientific literature points to vaccine hesitancy as being a major health concern. During time of pandemics, increased consciousness of health behaviors may be encountered. The current study aimed to assess attitudes to general vaccines and to COVID-19 vaccines in particular among adult (>18) Israeli general public, and among Israeli dentists and dental hygienists. Cross-sectional surveys were filled out by a total of 501 participants (361 Israeli adults >18 years, 73 dental hygienists, and 67 dentists). Along with basic demographics, participants responded to the Hebrew VAX, COVID-VAX and HCS scales. Group comparisons were analyzed using *t* tests and ANOVAs with Scheffe’s test used for post hoc comparisons. Dental hygienists demonstrated significantly higher anti-vaccinations approaches than both dentists (*p* < 0.01) and the general public (*p* < 0.05). In all groups, attitudes towards the COVID-19 vaccines were more negative compared to attitudes towards general vaccines, with hygienists demonstrating significant negative attitudes compared to dentists (*p* < 0.05). The general public (*p* = 0.56) and hygienists demonstrated increased health awareness compared to dentists (*p* < 0.05). As health awareness has increased during the COVID-19 pandemic primary strategies to combat vaccine hesitancy should be implemented in the general public, and in particular, an dental teams.

## 1. Introduction

Since its outbreak in December 2019, severe acute respiratory syndrome coronavirus-2 (SARS-CoV-2), the virus responsible for the COVID-19 pandemic, has considerably affected the worldwide population [1]. Health authorities and the medical community identify vaccines as an effective tool for managing public health [2] and, accordingly, long-term control of the COVID-19 pandemic will be determined by preventive vaccines [3]. Since the early discovery of vaccinations by Eduard Jenner in 1796, vaccines have played a key role in combating and controlling various highly infectious diseases such as polio, hepatitis B, and influenza viruses [4]. Since the SARS-CoV-2 viral sequence was published in January of 2020, multiple laboratories have worked at an accelerated pace on candidate vaccines against the SARS-CoV-2; these trials are ongoing. Based on preliminary data showing that the efficacy of the BNT162b2 vaccine was 95% in persons 16 years and above [5], several countries, including Israel, have begun to vaccinate their populations.

As recently published, the efficacy of the COVID-19 vaccination programs in Israel using the BNT162b2 mRNA vaccine appears to be high across different age groups, albeit with some lower efficacy in those with multiple coexisting conditions [6]. This seems highly promising as the death toll from SARS-CoV-2 infection has passed 6000 deaths since March, 2020 [7].

The World Health Organization (WHO) has classified healthcare workers (HCWs) as a priority group for COVID-19 vaccination [8]. In particular, COVID-19 has been reported to be airborne by aerosols generated during dental treatments, resulting in potential risks of infection for dental staff and patients [9]. Therefore, it has been noted that dental teams should be especially careful to ensure a safe environment for themselves and patients [10].

On 19 December 2020, Israel began vaccinating healthcare staff, including dental staff, along with people >65 years of age and people at high risk of the complications of COVID-19 (e.g., having severe lung or heart disease) [11]. By 4 January 2021, Israel was ranked as the country with the highest per capita COVID-19 vaccination rate [12]. Despite this, prior research has shown that the global phenomenon of vaccine hesitancy includes segments of the population in Israel [13]. Thus, in the current study, we sought to examine the attitudes of the Israeli adult population regarding vaccinations in general, and the COVID-19 vaccinations in particular, compared to dental staff attitudes (i.e., dentists and dental hygienists) regarding such vaccinations.

Scientific literature indicates that vaccine hesitancy has increased since the influenza pandemic in 2009 [14]. Vaccine hesitancy may exist even among people who believe in the importance of vaccinations [15], and in 2019 was declared as one of the top ten threats to global health by the WHO [16]. Vaccine hesitancy has multiple causes including lack of knowledge, lack of awareness, past experiences, perceived importance of vaccinations, subjective norms, religious and moral convictions, mistrust of those involved in producing and selling vaccinations, and risk perception [14,17]. Of importance to the current study, the global COVID-19 vaccines’ accelerated development and testing may raise apprehensions about safety, therefore, increasing hesitancy toward the COVID-19 vaccination [18].

Vaccine hesitancy is not limited to the general public; despite recommendations for the vaccination of all HCWs against infectious diseases [19], HCWs also report vaccine hesitancy [14,20,21]. Vaccine hesitancy among HCWs is also influenced by multiple factors including fear of side-effects [21] and misconceptions concerning safety and importance, as demonstrated among an Italian dental team [22]_._ Individual beliefs and perceptions, embedded in personal, social, and cultural values, indirectly affect people’s health behaviors, frequently without their awareness [23,24], which can be seen in decisions about vaccinations. Rosenstock’s health belief model (HBM) [25] may explain awareness of health behaviors concerning vaccinations. According to the HBM, health behaviors are predicted by perceived susceptibility to and severity of a particular health problem, perceived benefits of the recommended behavior to decrease risk or gravity of the health problem, and perceived barriers to implementing the behavior [26]. A recent meta-analysis showed that perceived benefits and barriers are the most substantial predictors of health behavior [27]. Concerning vaccinations, critical review of the data suggests that perceptions of low risk and severity are associated with vaccine hesitancy [14] and this model has also been found to meaningfully predict intentions to receive COVID-19 vaccination [28]. Regarding health awareness, elevated levels have been observed during the COVID-19 pandemic [29].

The present study used the risk perception theory as its foundation, described in a recent meta-analysis [30]. This theory overlaps with the HBM, and relates to perceived vulnerability, likelihood, and harm associated with an outcome (illness). Based on the risk perception theory, concern over possible side-effects should play a substantial role in vaccine hesitancy [17]. In addition, expressed doubts and contradictory information may impede the process of making rational decisions regarding vaccines. Presently, the spread of such information may be escalated by the social media and other evolving ways of communication [17]. Decision-making may even deteriorate further during times of pandemics, such as COVID-19 [31]. As previously stated [31], individualized views, personal experience, social factors along with connection with close environments, may exert significant effects on risk perception during COVID-19 pandemic.

Pandemic outbreaks may trigger the human body to respond by adjusting its physiological, physical and mental health states. A proposed mechanism for human interactions at times of pandemics may be supported by the behavioral immune system (BIS) theory [32]. With relation to the COVID-19 pandemic, the BIS theory suggests that viral pandemics may induce a cascade of emotional and cognitive reactions within an individual, which leads to an aversive type of behavior in order to avoid contraction of the virus [33]. That is, when a viral agent is in the surroundings, subsequent avoidant behaviors may ensue to avoid contraction of such virus, e.g., SARS-CoV-2; the latter may even manifest in refusal to get vaccinated against it. In accordance, it may be that during times of pandemics, increased awareness of health behaviors may be encountered.

Based on the above theories and findings, the current study aimed to examine attitudes regarding vaccinations and awareness of health behaviors within the adult Israeli population (>18 years) and among Israeli dentists and dental hygienists during the COVID-19 pandemic. As most Israeli dental hygienists are female, and to eliminate gender bias, differentiation between dentists and hygienists was performed. This is in accordance with previous findings demonstrating negative vaccination attitudes among female nurses in Israeli hospitals [18]. In addition, we aimed to assess possible differences between general attitudes toward vaccines and the attitudes toward the COVID-19 vaccine specifically. It was first hypothesized that dental hygienists would show higher anti-vaccinations attitudes than either dentists or the adult sample, as previously noted [18]. The second hypothesis was that attitudes for each of the three groups regarding the COVID-19 vaccination would be more negative than attitudes regarding vaccines generally. Finally, regarding health behaviors awareness, it was hypothesized that during times of the COVID-19 pandemic, an increased awareness would be exhibited.

## 2. Materials and Methods

### 2.1. Sampling and Procedure

Ethical approval for the current study was granted by the Institutional Review Board of the authors’ university (M.S., L.G.-K., Y.H.-R., M.B.-E.). An online platform (www.imkforms.com (accessed on 22 December 2020)) was used to conduct the survey. Potential participants were approached via social media (WhatsApp, Facebook), along with personal contacts and subsequent snowball sampling, all of which included a description of the study and its objectives, along with an assurance of anonymity. Each participant then signed an electronic informed consent. From 22 December 2020 to 1 January 2021, data were collected from 501 Israeli participants (361 Israeli adults >18 years, 73 dental hygienists, and 67 dentists).

### 2.2. Measurements

The following basic demographics were collected: Age (in years), gender (coded as ‘1 = male’, ‘2 = female’), relationship status (coded as ‘1 = not being in a committed relationship’, ‘2 = being in a committed relationship’), and degree of religiosity (coded as ‘1 = secular’, ‘2 = traditional’, ‘3 = orthodox’).

In addition, the following self-reported measures were used:

### 2.3. Vaccination Attidues Examination Scale (VAX)

The VAX scale is a 12-item scale (including four subscales) used to assess anti-vaccination attitudes [29]. Each item is scored on a scale of ‘1 = strongly disagree’ to ‘6 = strongly agree’. Previous studies indicate high internal consistency [34,35]. In this study, the VAX was translated to Hebrew by the authors (back-translation verified by a native English speaker, L.G.K), and showed excellent internal consistency (Cronbach’s α = 0.93, consistent with prior studies). To assess attitudes specifically regarding COVID-19 vaccines, we modified each of the 12 items to specifically address COVID-19 vaccines, with the modified scale labeled “COVID-VAX”. The latter’s internal consistency was also shown to be high (Cronbach’s α = 0.93). In the current study we used both the VAX and the COVID-VAX scales. A higher total score, for both scales, indicates more negative attitudes toward vaccinations. The VAX scales may be further sub-categorized based on items number: items #1–3 relate to mistrust of vaccine benefits, #4–6 to worries over unforeseen future effects, #7–9 to concerns about commercial profits, #10–12 to preference for natural immunity [34].

### 2.4. Health Consciousness Scale (HCS)

The HCS scale was deployed in the current study to evaluate participants’ awareness of health behaviors [36], e.g., “I’m alert to changes in my health”. The 9 items were translated to Hebrew by the authors (back-translation verified by a native English speaker, L.G.K). The scale is coded as ‘1 = totally disagree’ to ‘5 = totally agree’. A higher total score indicates greater awareness of health behaviors. Cronbach’s α for the translated HCS was 0.87, indicating very good internal consistency.

### 2.5. Statistical Analyses

Descriptive data were used to describe the characteristics of the sample, both in terms of demographics and scale scores; internal consistency of the scale scores was evaluated with Cronbach’s alpha. Group comparisons were done using *t* tests and analyses of variance (ANOVA) with the Scheffe’s test used for post hoc comparisons, with level of significance set at α ≤ 0.05. In addition, a multivariate analysis of covariance (MANOVA) was conducted utilizing VAX, COV-VAX and their sub-items and HCS items as dependent variables, with age, sex, marital status, degree of religiosity, and occupation (i.e., dentists, hygienists, or general public) as independent variables (see Supplementary File S1, Table S1). Analyses were conducted using SPSS version 25 (IBM, Armonk, NY, USA).

## 3. Results

Basic demographics are summarized in Table 1. ANOVAs revealed that there were mean group differences for total VAX scores (*F* = 5.074, *p =* 0.007), total COVID-VAX scores (*F* = 4.43, *p =* 0.012) and HCS scores (*F* = 4.502, *p =* 0.012). For all variables, dentists and hygienists were significantly different from one another whereas dentists and the general public were not. More specifically, dental hygienists had more negative attitudes toward the vaccines generally as compared to both other groups. Hygienists demonstrated significant mistrust of vaccine benefits (M = 7.05 ± 3.71) compared to dentists (M = 5.00 ± 2.29, *p* = 0.003), and near-significant differences from the general public. Hygienists also demonstrated more worries over unforeseen future effects (M = 11.39 ± 3.51) compared to dentists (M = 9.16 ± 4.00, *p =* 0.004) and the general public (M = 9.20, *p* = 0.001). Hygienists also had more negative attitudes toward the COVID-19 vaccines and scored higher on health consciousness than did dentists (see Table 2), while exhibiting differences in different sub-scales of the COVID-VAX subscales, compared to the VAX sub-scales previously mentioned. Hygienists seemed to differ in a near-significant manner (M = 8.53 ± 3.85) from dentists in their mistrust of COVID-19 vaccines’ benefits (M = 6.82 ± 3.57, *p* = 0.052), while showing significantly more concerns over commercial profiteering (M = 6.34 ± 3.74) compared to dentists (M = 4.62 ± 2.90, *p =* 0.027). Regarding natural immunity, hygienists demonstrated significantly higher preference (M = 7.38 ± 3.33) compared to dentists (M = 5.46 ± 3.23, *p =* 0.009). As dental hygienists were all female, additional ANOVAs analyses were conducted based on gender (see Table 3). As noted, there are significant differences in mistrust of vaccine benefits between hygienists and female dentists, and worries about unforeseen side-effects between hygienists and females in the adult population. In addition, hygienists seemed to demonstrate a higher anti-vaccination approach compared to the female adult population.

Regarding differences between VAX and COVID-VAX scores, *t* tests indicated that all groups had more negative attitudes toward the COVID-19 vaccine than toward vaccines in general (See Table 4).

MANOVA analysis results revealed that degree of religiosity was not significantly associated with the different dependent variables, hence it was omitted from Table S1. As can be seen, age was associated with COV-VAX sub-items #1–3 (*F* = 9.57; *p* < 0.01; partial η^2^ = 0.019). Gender was associated with VAX and COV-VAX sub-items #10–12 (*F* = 5.58; *p* < 0.05; and partial η^2^ = 0.011; *F* = 3.95; *p* < 0.05; partial η^2^ = 0.008, respectively). Marital status was significantly associated with VAX scale (*F* = 10.53; *p* < 0.001; partial η^2^ = 0.021) along with its sub-items (*F* = 4.86; *p* < 0.05; partial η^2^ = 0.010; F = 8.81; *p* < 0.01; partial η^2^ = 0.018; *F* = 5.51; *p* < 0.05; partial η^2^ = 0.011; and *F* = 10.03; *p* < 0.01; partial η^2^ = 0.020). Marital status was also associated with COV-VAX scale (*F* = 7.55; *p* < 0.01; partial η^2^ = 0.021) and #7–9 (*F* = 5.58; *p* < 0.05; partial η^2^ = 0.012) and #10–12 sub-items (*F* = 13.66; *p* < 0.001; partial η^2^ = 0.027).

## 4. Discussion

To the best of our knowledge, the present study is the first to compare attitudes regarding vaccine hesitancy and awareness of health behaviors in the adult Israeli population, Israeli dentists, and dental hygienists during the COVID-19 pandemic. Consistent with the first hypothesis, hygienists reported greater COVID-19 vaccine hesitancy than dentists. This result may be surprising as COVID-19 is airborne by aerosols generated in dental treatments, thereby putting dental staff at high risk of occupational exposure to COVID-19 [9], and may have been expected to prompt a positive predisposition to a vaccine that could mitigate this risk. Dentists demonstrated less hesitancy compared to hygienists, in accordance with previous studies that reported doctors to be more willing to be vaccinated against COVID-19 than other HCWs [37,38]. Our results are in accordance with an Israeli study done one week after the first implementation of social distancing and quarantine regulations during the pandemic [18]. The researchers reported the highest anti-vaccination attitudes among nurses, thereafter the general public, while doctors reported the highest willingness to be vaccinated [18]. Another possible explanation lies in previously identified gender differences in vaccine hesitancy, with men being less hesitant than women [18]. The inherent male inclination for risk-taking may explain this difference [39]. In the current study, 65.7% of the dentists were male, whereas the dental hygienists were all female, thus making gender a likely contributor to the observed differences. However, as noted in Table 3, gender differences remained significant especially with hygienists demonstrating a higher anti-vaccination approach compared to female counterparts included in the current study. A possible explanation may be that females tend to be more aware of health behaviors, e.g., fertility issues, and thus may have increased vaccine hesitancy. In addition, most of the study’s participants indicated being in a committed relationship. As demonstrated by previous studies, marital status may have an effect on vaccine hesitancy, with single parents [40,41] or those divorced [42] demonstrating increased vaccine hesitancy.

In line with the second hypothesis, attitudes regarding the COVID-19 vaccination were more negative compared to attitudes regarding other vaccines for all three groups. This finding is partially in line with previous findings. For example, the rate of agreeing to a COVID-19 vaccine among Israeli physicians and nurses was lower than that for the seasonal influenza vaccination [18]. In other studies, 25% of Americans and 20% of Canadians stated they would reject a SARS-CoV2 vaccine, a stance that was associated with a generally negative attitude toward vaccinations [43], and among nurses in Hong Kong, the main barrier to the COVID-19 vaccine was suspicion about safety, efficacy, and effectiveness, while the main barrier to the flu vaccine was doubt whether it was needed [44]. The results of the current finding may be understood in light of fears related to the rapid testing and approval process of the COVID-19 [18]. Specifically, the differences between attitudes towards vaccines in general and the COVID-19 vaccine in particular may be partially explained by the rapid manner in which the COVID-19 vaccine was produced, which may trigger vaccine hesitancy among the general population as well as dental care providers. Despite the promotion of the COVID-19 vaccine’s efficacy and safety by institutions [45,46], the internet and social media such as Facebook, Twitter, and YouTube provide anti-vaccination activists an opportunity to advance their skeptical and usually negative messages [47,48,49,50].

Considering the newness of the COVID-19 pandemic, its rapid global dissemination, the fast trajectories of testing of SARS-CoV-2 vaccines, the partial lack of transparency about effectiveness rates, and the continuously changing claims about effectiveness, it may be considered rational to be hesitant, to wait and see, etc. Hesitancy may be about trustworthiness [51]. Hesitance may be also about waiting until claims about safety and effectiveness become more robust and credible. Such factors may play a crucial role in both the dental care providers and the general public.

In line with the third hypothesis, and according to the HBM model [28,29], it may be that both hygienists and the general public deem it necessary to increase their health awareness, as commercial mistrust and preference for natural immunity were stronger motives to disengage in vaccination rather than the previously mentioned positive predictors.

The present study has several strengths and limitations. The study was based on a cross-sectional design; hence, causality cannot be concluded from the findings. The number of participants in the study was moderate, with the time interval being short. These may serve as limitations. As the long-term effects of the COVID-19 vaccine are still unknown, a longitudinal study that examines the attitudes and behaviors towards the COVID-19 vaccine in the general public, and in dental teams in particular, is recommended. In addition, the study sample was based on an online design; therefore, there may be bias towards people with technological knowledge. As stated in the VAX scale instructions, its scoring is based on averages. There are no categories that specify at what average/score one may be considered as vaccine hesitant. Therefore, no calculations of vaccine hesitancy frequency were conducted in the current study. Nevertheless, this appears to be the first study to examine COVID-19 vaccine hesitancy and awareness of behaviors in a dental context since the initiation of the COVID-19 vaccination program in Israel. 

HCWs, dentists included, maintain a central role in promoting vaccines, as their recommendations are vital for vaccine acceptance among the general public [52]. Just as dentists and dental hygienists have been found to value their role in promoting the HPV vaccine [53] they should recognize the important role they have in promoting the COVID-19 vaccine in order to protect themselves and their patients. Nonetheless, dental care professionals may be prone to stigma and discrimination based on vaccination attitudes during the COVID-19 pandemic [54,55]. Our data implies that dental hygienists’ possess higher anti-vaccination attitudes during such times, and may be prone to negative stigma and discrimination based on such attitudes. Therefore, coping strategies may be provided for this group, as described elsewhere [54].

It has also been suggested that a COVID-19 vaccination “passport” be provided with added benefit/s to the vaccinated, with the aim of encouraging the general adult population to adhere to COVID-19 vaccine recommendations [38].

The current study was held during the COVID-19 pandemic and during the COVID-19 vaccination programs in Israel. As described in previous studies, vaccine hesitancy may be affected by specific contexts, i.e., the COVID-19 pandemic, as well as the specific national history of vaccination, specific national problems [56,57], level of income and education [58], care for family well-being [59], and adherence to social guidelines and restrictions [60]. Thus, such unique factors may inform interpretation of the current study’s results.

## 5. Conclusions

The findings and insights in the present study are essential, as they suggest primary targets to combat vaccine hesitancy, such as mistrust of benefits and worries over unforeseen side-effects as the in case of general vaccines; and commercial concerns and preference for natural immunity in the case of COVID-19 vaccinations. Continuous campaigns, vaccination education programs, and promotion of trust by the local health authorities may aid in decreasing vaccine hesitancy among both dental care providers and the general public. Such actions and others were utilized during the COVID-19 vaccination programs in Israel, as summarized in Shilo et al. [61].

## Figures and Tables

**Table 1:** Basic demographics of the study sample (Israeli dentists, dental hygienists and adults >18 years (*n =* 501)).

Study Group	Number of Participants	Age (Mean (±SD))	Gender	Relationship Status	Degree of Religiosity
Dentists	67	42.13 (±10.66)	65.7% male, 34.3% female	C = 88.1%, NC = 11.9%	S = 77.6%, O = 13.4%, T = 9%
Dental hygienists	73	44.60 (±16.32)	100% female	C = 86.3% NC = 13.7%	S = 72.6%, T = 13.7%, O = 13.7%
General population	361	39.04 (±15.59)	69.52% female, 30.47% male	C = 76.5% NC = 23.5% relationship	S = 73.1% secular, T = 14.1% traditional, O = 12.7% orthodox

Notes: C = In a committed relationship, NC = not in a committed relationship, S = Secular, T = Traditional, O = Orthodox.

**Table 2:** ANOVA results indicating differences among the study participants based on the study variables, using post hoc Scheffé tests.

Factors	Dentists (*n* = 67), 95% CI	Dental Hygienists (*n* = 73), 95% CI	Adult Population (*n* = 361), 95% CI	*F*	Post hoc Scheffé
VAX score M (±SD)	25.19 (±9.59), 22.85–27.53	31.47 (±9.76), 29.20–33.75	27.48 (±12.86), 26.15–28.81	5.07 **	1 < 2 **, 2 > 3 *
COVID-VAX score M (±SD)	26.53 (±9.42), 24.23–28.83	32.84 (±10.95), 30.29–35.40	29.91 (±13.30), 28.53–31.29	4.43 *	1 < 2 *
HCS score M (±SD)	32.38 (±5.27), 31.10–33.67	35.15 (±6.01), 33.74–36.55	34.17 (±5.51), 33.60–34.74	4.50 *	1 < 2 *
VAX items #1–3 (mistrust of vaccine benefit)	5.00 (±2.29), 4.43–5.56	7.05 (±3.71), 6.18–7.92	6.16 (±3.75), 5.77–6.54	5.74 **	1 < 2 **
VAX items #4–6 (worries over unforeseen future effects)	9.16 (±4.00), 8.18–10.14	11.39 (±3.51), 10.57–12.21	9.20 (±3.99), 8.78–9.61	9.76 ***	1 < 2 **, 2 > 3 ***
VAX items #7–9 (concerns about commercial profits)	4.47 (±2.60), 3.84–5.11	5.49 (±2.73), 4.85–6.13	5.22 (±3.46), 4.86–5.58	1.90	
VAX items #10–12 (preference to natural immunity)	6.55 (±3.29), 5.74–7.35	7.53 (±3.47), 6.72–8.34	6.90 (±3.85), 6.50–7.30	1.30	
COVID-VAX items #1–3	6.82 (±3.57), 5.94–7.69	8.53 (±3.85), 7.63–9.43	7.85 (±4.30), 7.41–8.30	3.03 *	
COVID-VAX items #4–6	9.62 (±3.70), 8.72–10.53	10.58 (±3.39), 9.79–11.38	9.88 (±3.93), 9.47–10.29	1.31	
COVID-VAX items #7–9	4.62 (±2.90), 3.91–5.33	6.34 (±3.74), 5.46–7.21	5.62 (±3.89), 5.21–6.02	3.68 *	1 < 2 *
COVID-VAX items #10–12	5.46 (±3.23), 4.67–6.25	7.38 (±3.33), 6.60–8.16	6.55 (±3.83), 6.15–6.95	4.75 **	1 < 2 **

Notes: 1 = Dentists, 2 = Dental hygienists, 3 = Adult population, CI = Confidence interval, VAX score = vaccination attitudes, COVID-VAX score = COVID-19 vaccination attitudes, HCS score = awareness of health behaviors. * *p* ≤ 0.05; ** *p* ≤ 0.01; *** *p* ≤ 0.001.

**Table 3:** ANOVA results indicating differences among the female study participants (*n* = 347) based on the study variables, using post hoc Scheffé tests.

Factors	Dentists (*n* = 23), 95% CI	Dental Hygienists (*n* = 73), 95% CI	Adult Population (*n* = 251), 95% CI	*F*	Post hoc Scheffé
VAX score M (±SD)	24.65 (±6.91), 21.66–27.64	31.47 (±9.76), 29.20–33.75	27.17 (±13.43), 25.50–28.84	4.25 *	2 > 3
COVID-VAX score M (±SD)	27.13 (±5.78), 24.62–29.63	32.84 (±10.95), 30.29–35.40	29.94 (±13.93), 28.20–31.67	2.18	
HCS score M (±SD)	32.43 (±3.76), 30.80–34.06	35.15 (±6.01), 33.74–36.55	34.46 (±5.62), 33.76–35.16	2.05	
VAX items #1–3 (mistrust of vaccine belief)	4.73 (±2.17), 3.79–5.68	7.05 (±3.71), 6.18–7.92	6.19 (±3.94), 5.70–6.69	3.44 *	1 < 2
VAX items #4–6 (worries over unforeseen future effects)	9.04 (±3.86), 7.37–10.71	11.39 (±3.51), 10.57–12.21	9.09 (±4.16), 8.57–9.61	9.49 **	2 > 3
VAX items #7–9 (concerns about commercial profits)	4.39 (±2.18), 3.44–5.33	5.49 (±2.73), 4.85–6.13	5.31 (±3.59), 4.87–5.76	0.96	
VAX items #10–12 (preference to natural immunity)	6.47 (±2.44), 5.42–7.53	7.53 (±3.47), 6.72–8.34	6.55 (±3.79), 6.08–7.02	2.08	
COVID-VAX items #1–3	6.91 (±3.30), 5.48–8.34	8.53 (±3.85), 7.63–9.43	8.01 (±4.44), 7.46–8.56	1.29	
COVID-VAX items #4–6	10.13 (±2.68), 8.96–11.29	10.58 (±3.39), 9.79–11.38	10.01 (±4.10), 9.50–10.52	0.62	
COVID-VAX items #7–9	4.43 (±1.75), 3.67–5.19	6.34 (±3.74), 5.46–7.21	5.67 (±4.10), 5.16–6.18	2.17	
COVID-VAX items #10–12	5.65 (±2.70). 4.48–6.82	7.38 (±3.33), 6.60–8.16	6.23 (±3.73), 5.77–6.70	3.45 *	

Notes: 1 = Dentists, 2 = Dental hygienists, 3 = Adult population, CI = Confidence interval, VAX score = vaccination attitudes, COVID-VAX score = COVID-19 vaccination attitudes, HCS score = awareness of health behaviors. * *p* ≤ 0.05; ** *p* ≤ 0.001.

**Table 4:** Differences between VAX and COVID-VAX scores among the different participants groups in the current study.

	VAX Score M (±SD)	COVID-VAX Score M (±SD)	*t-*Statistic	Sig.
Dentists (*n* = 67)	25.19 (±9.59)	26.53 (±9.42)	−2.113	0.038
Dental hygienists (*n* = 73)	31.47 (±9.76)	32.84 (±10.95)	−2.290	0.025
Adult population (*n* = 361)	27.48 (±12.86)	29.91 (±13.30)	−7.655	0.001

Notes: VAX score = vaccination attitudes, COVID-VAX score = COVID-19 vaccination attitudes.

## Data Availability

No new data were created or analyzed in this study. Data sharing is not applicable to this article.

## Supplementary Materials

The following are available online at https://www.mdpi.com/article/10.3390/ijerph18073804/s1, Table S1: MANOVA results including effect size for HCS, VAX, COV VAX scales along with their sub items (*n*= 501). File S1: The Vaccination Attitudes Examination (VAX) Scale.
