# Supplementary material for: Increased COVID-19 Vaccination Hesitancy and Health Awareness amid COVID-19 Vaccinations Programs in Israel
Source: Int J Environ Res Public Health. 2021 Apr 6;18(7):3804. doi: 10.3390/ijerph18073804 (PMC8038659; doi:10.3390/ijerph18073804)
Supplement: Supplementary file 1 [file ijerph-18-03804-s001.zip › ijerph-1136554-supplementary- final/File S1.pdf]

The Vaccination Attitudes Examination (VAX) Scale

Martin, L.R., & Petrie, K.J. (2017). Understanding the dimensions of anti-vaccination attitudes: The Vaccination Attitudes Examination (VAX) Scale. *Annals of Behavioral Medicine*. doi: 10.1007/s12160-017-9888-y

These questions are designed to help us better understand people's beliefs about vaccinations. Please mark the choices that most accurately reflect your feelings or beliefs. There are no right or wrong responses.

I feel safe after being vaccinated. (-)	Strongly Disagree 1	2	3	4	5	Strongly Agree 6
I can rely on vaccines to stop serious infectious diseases. (-)	Strongly Disagree 1	2	3	4	5	Strongly Agree 6
I feel protected after getting vaccinated. (-)	Strongly Disagree 1	2	3	4	5	Strongly Agree 6
Although most vaccines appear to be safe, there may be problems that we haven't yet discovered.	Strongly Disagree 1	2	3	4	5	Strongly Agree 6
Vaccines can cause unforeseen problems in children.	Strongly Disagree 1	2	3	4	5	Strongly Agree 6
I worry about the unknown effects of vaccines in the future.	Strongly Disagree 1	2	3	4	5	Strongly Agree 6
Vaccines make a lot of money for pharmaceutical companies, but don't do much for regular people.	Strongly Disagree 1	2	3	4	5	Strongly Agree 6
Authorities promote vaccination for financial gain, not for people's health.	Strongly Disagree 1	2	3	4	5	Strongly Agree 6
Vaccination programs are a big con.	Strongly Disagree 1	2	3	4	5	Strongly Agree 6
Natural immunity lasts longer than a vaccination.	Strongly Disagree 1	2	3	4	5	Strongly Agree 6
Natural exposure to viruses and germs gives the safest protection.	Strongly Disagree 1	2	3	4	5	Strongly Agree 6
Being exposed to diseases naturally is safer for the immune system than being exposed through vaccination.	Strongly Disagree 1	2	3	4	5	Strongly Agree 6

After reverse-coding, scale and subscale scores are created by averaging the relevant items (1, 2, 3 = mistrust of vaccine benefit; 4, 5, 6 = worries over unforeseen future effects; 7, 8, 9 = concerns about commercial profiteering; 10, 11, 12 = preference for natural immunity).
