# Supplementary material for: Increased COVID-19 Vaccination Hesitancy and Health Awareness amid COVID-19 Vaccinations Programs in Israel
Source: Int J Environ Res Public Health. 2021 Apr 6;18(7):3804. doi: 10.3390/ijerph18073804 (PMC8038659; doi:10.3390/ijerph18073804)
Supplement: Supplementary file 1 [file ijerph-18-03804-s001.zip › ijerph-1136554-supplementary- final/ijerph-1136554-Table S1.doc]

Online Supporting Martial
Table S1. MANOVA results including effect size for HCS, VAX, COV-VAX scales along with their sub-items (n = 501).
Source	DV	Type III Sum of Squares	Mean Square	F	Sig.	Partial Eta Squared	
Age	VAX items #1-3 (mistrust of vaccine belief)	.453	.453	.036	.850	.000	
	VAX items #4-6 (worries over unforeseen future effects)	18.238	18.238	1.193	.275	.002	
	VAX items #7-9 (concerns about commercial profits)	.375	.375	.035	.851	.000	
	VAX items #10-12 (preference to natural immunity)	1.550	1.550	.114	.736	.000	
	COVID-VAX items #1-3	161.071	161.071	9.571	.002	.019	
	COVID-VAX items #4-6	.151	.151	.010	.919	.000	
	COVID-VAX items #7-9	15.469	15.469	1.104	.294	.002	
	COVID-VAX items #10-12	11.372	11.372	.860	.354	.002	
	HCS score	36.492	36.492	1.182	.277	.002	
	VAX score	31.099	31.099	.218	.641	.000	
	COVID-VAX score	24.976	24.976	.161	.689	.000	
Gender	VAX items #1-3 (mistrust of vaccine belief)	.825	.825	.065	.799	.000	
	VAX items #4-6 (worries over unforeseen future effects)	7.062	7.062	.462	.497	.001	
	VAX items #7-9 (concerns about commercial profits)	7.566	7.566	.715	.398	.001	
	VAX items #10-12 (preference to natural immunity)	75.918	75.918	5.587	.018	.011	
	COVID-VAX items #1-3	22.930	22.930	1.363	.244	.003	
	COVID-VAX items #4-6	21.807	21.807	1.485	.224	.003	
	COVID-VAX items #7-9	1.629	1.629	.116	.733	.000	
	COVID-VAX items #10-12	52.241	52.241	3.951	.047	.008	
	HCS score	60.123	60.123	1.948	.163	.004	
	VAX score	59.471	59.471	.417	.519	.001	
	COVID-VAX score	12.298	12.298	.079	.779	.000	
Marital status	VAX items #1-3 (mistrust of vaccine belief)	61.861	61.861	4.865	.028	.010	
	VAX items #4-6 (worries over unforeseen future effects)	134.749	134.749	8.815	.003	.018	
	VAX items #7-9 (concerns about commercial profits)	58.353	58.353	5.515	.019	.011	
	VAX items #10-12 (preference to natural immunity)	136.394	136.394	10.038	.002	.020	
	COVID-VAX items #1-3	38.015	38.015	2.259	.133	.005	
	COVID-VAX items #4-6	31.129	31.129	2.120	.146	.004	
	COVID-VAX items #7-9	82.482	82.482	5.886	.016	.012	
	COVID-VAX items #10-12	180.689	180.689	13.666	.000	.027	
	HCS score	18.394	18.394	.596	.441	.001	
	VAX score	1504.743	1504.743	10.539	.001	.021	
	COVID-VAX score	1174.360	1174.360	7.552	.006	.015	
Occupation	VAX items #1-3 (mistrust of vaccine belief)	112.890	56.445	4.439	.012	.018	
	VAX items #4-6 (worries over unforeseen future effects)	310.010	155.005	10.140	.000	.039	
	VAX items #7-9 (concerns about commercial profits)	20.240	10.120	.956	.385	.004	
	VAX items #10-12 (preference to natural immunity)	78.210	39.105	2.878	.057	.012	
	COVID-VAX items #1-3	64.229	32.115	1.908	.149	.008	
	COVID-VAX items #4-6	21.633	10.816	.737	.479	.003	
	COVID-VAX items #7-9	73.990	36.995	2.640	.072	.011	
	COVID-VAX items #10-12	165.163	82.581	6.246	.002	.025	
	HCS score	160.119	80.060	2.594	.076	.010	
	VAX score	1505.328	752.664	5.272	.005	.021	
	COVID-VAX score	1096.792	548.396	3.527	.030	.014	
Notes: Occupation (Dentists, hygienists, general public); *p ≤ .05; **p ≤ .01; ***p ≤ .001.
