# Supplementary figures and images for: Increased COVID-19 Vaccination Hesitancy and Health Awareness amid COVID-19 Vaccinations Programs in Israel
Source: Int J Environ Res Public Health. 2021 Apr 6;18(7):3804. doi: 10.3390/ijerph18073804 (PMC8038659; doi:10.3390/ijerph18073804)

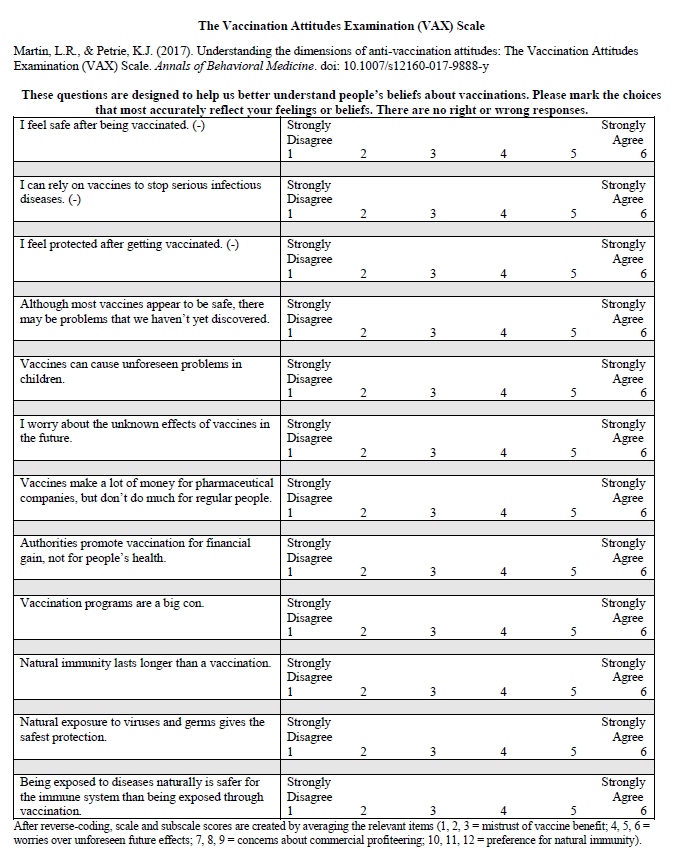

Supplement: Supplementary file 1 [file ijerph-18-03804-s001.zip › ijerph-1136554-supplementary- final/File S1.docx]
